# Effects of Exogenous Spermidine on Seed Germination and Physiological Metabolism of Rice Under NaCl Stress

**DOI:** 10.3390/plants13243599

**Published:** 2024-12-23

**Authors:** Xiaohui Yang, Jian Xiong, Xiaole Du, Minmin Sun, Linchong Ding, Wanqi Mei, Zhiyuan Sun, Naijie Feng, Dianfeng Zheng, Xuefeng Shen

**Affiliations:** 1 College of Coastal Agricultural Sciences, Guangdong Ocean University, Zhanjiang 524008, China; 2112204049@stu.gdou.edu.cn (X.Y.); 2112204010@stu.gdou.edu.cn (J.X.); duxiaole2022@163.com (X.D.); 2112104037@stu.gdou.edu.cn (M.S.); dinglinchong0405@163.com (L.D.); meiwqcn@outlook.com (W.M.); sunzhiyuan@hndx2.wecom.work (Z.S.); fengnj@gdou.edu.cn (N.F.); zhengdf@gdou.edu.cn (D.Z.); 2 National Saline-Tolerant Rice Technology Innovation Center, South China, Zhanjiang 524008, China

**Keywords:** rice, NaCl stress, spermidine, seed germination, seedling growth

## Abstract

Salt stress is one of the principal abiotic stresses limiting agricultural production and seriously inhibiting seed germination rates. This study selected the salt-tolerant rice variety HD961 and the salt-sensitive rice variety 9311 as experimental materials to investigate the physiological and metabolic effects of exogenous Spd seed priming on rice seeds and seedlings under NaCl stress. The experiment involved treating rice seeds with 0.1 mmol·L^−1^ Spd and then subjecting them to 100 mmol·L^−1^ NaCl stress for 24 h, with sampling for analysis at the 24 h and the four-leaf-one-heart stage. The results indicated that under NaCl stress, the rice’s germination and vigor indices significantly decreased. However, exogenous Spd seed priming reduced the accumulation of malondialdehyde, enhanced the capacity for osmotic adjustment, and increased the amylase and antioxidant activity by 50.07% and 26.26%, respectively. Under NaCl stress, the morphological development of rice seedlings was markedly inhibited, whereas exogenous Spd seed priming improved the aboveground and belowground biomass of the rice under stress conditions, as well as the content of photosynthetic pigments. It also reduced the damage to seedlings from electrical conductivity, helped maintain ionic balance, and promoted the excretion of Na^+^ and Cl^−^ and the absorption of K^+^ and Ca^2+^. In the salt-sensitive rice variety 9311, the soluble protein content increased by 15.12% compared to the salt-tolerant rice variety HD961, especially under 100 mmol·L^−1^ NaCl stress, when the effect of exogenous Spd seed priming was more pronounced. In summary, these findings might provide new research perspectives and strategies for improving the salt tolerance of rice under NaCl stress.

## 1. Introduction

Rice (*Oryza sativa* L.), a critical global food crop, provides a staple diet for over half of the world’s population. With societal progress, rice cultivation has shifted from traditional nursery transplanting to direct seeding [1], reducing labor and time costs. However, direct seeding requires higher-quality seeds. Seed germination, the inception of the plant’s life cycle, involves complex physiological changes [2], including energy production, starch transformation, and substance degradation [3], which are essential for rice growth [4]. Enhancing seed germination rates and seedling growth is crucial for improving seedling survival rates and promoting sustainable production [5,6,7]. The transition from seed germination to seedling growth is a vulnerable period in rice development, susceptible to adverse conditions such as NaCl stress [8], low temperatures [9], and waterlogging [10], which can severely disrupt normal growth and development. Research has indicated that using plant growth regulators for seed priming under stress conditions is a cost-effective method [11] capable of improving germination rates, promoting seedling growth, and thereby increasing yield.

Sodium chloride stress is a complex abiotic stress that severely affects the growth of major food crops such as rice, wheat, and sorghum [12,13]. Under NaCl stress, seed starch degradation is hindered due to reduced amylase activity [14,15], leading to decreased energy supply and consequently lower seed germination rates. Additionally, NaCl stress causes an excessive accumulation of sodium and chloride ions and the extrusion of potassium and calcium ions, leading to cellular ion imbalance [16]. Furthermore, NaCl stress delays water uptake, disrupts cellular structures, reduces seed germination and emergence rates, and extends the average germination time. The accumulation of hydrogen peroxide (H_2_O_2_) triggers the production of reactive oxygen species (ROS), disrupting the physiological metabolism of antioxidant enzymes and nonenzymatic antioxidants in rice seeds [17], affecting the biosynthesis and catabolism of abscisic acid (ABA) and gibberellins (GAs) [18], which negatively affects seed germination and seedling growth. Sodium chloride stress limits plant growth by reducing plant biomass and photosynthetic pigment content [19], affecting the functions of Photosystem I and Photosystem II in the chloroplasts, which are key to ROS synthesis [20]. Plants respond to stress by closing stomata and degrading chlorophyll, leading to increased osmotic stress capacity and electrolyte leakage (EL), weakened or halted photosynthesis, and subsequently reduced leaf transpiration rates and root water uptake capabilities [21], affecting nutrient absorption, causing plant nutrient imbalance, and reducing crop yield [22]. Therefore, enhancing seed germination tolerance under NaCl stress is crucial for successful seedling emergence, plant growth, and the maintenance of photosynthetic pigments [23].

Spermidine (Spd), a bioactive polyamine (PA) with intermediate activity, is ubiquitous in all living cells [24,25]. As a plant growth regulator, Spd acts as a signaling molecule in stress conditions, participating in plant growth and development and responses to biotic and abiotic stresses [26,27]. Studies have indicated that Spd’s protective role for plants is particularly pronounced under stress conditions, effectively mitigating damage from NaCl stress [28], temperature stress [3], and heavy-metal stress in rice. Furthermore, Spd can modulate plant hormones, promote root and bud growth, protect photosynthetic organs, scavenge ROS, and maintain redox balance [29]. Further research has found that Spd enhances the cold-stress resistance of wheat seeds by increasing the content of ABA and GAs [30]. Spermidine can also induce the expression of genes associated with NaCl stress, affect the synthesis of osmolytes, and reduce the levels of H_2_O_2_ and malondialdehyde (MDA) in seedlings [31,32]. In maize seedlings, Spd seed priming alleviates drought stress and enhances photosynthetic capacity [33]. Additionally, Spd increases the content of chlorophyll a and total chlorophyll, induces nitrate reductase activity, strengthens the antioxidant enzyme system, and improves the tolerance of tomatoes to NaCl stress [34].

Under NaCl stress conditions, the specific mechanisms by which Spd affects the physiological and biochemical processes in rice are not well understood [35,36]. For instance, exogenous Spd treatment can increase the content of proline and soluble sugars, which are involved in osmotic adjustment and enhancement of antioxidant enzyme activity, potentially aiding in the osmotic balance of rice under salt stress [37]. The differences in these effects among different salt-tolerant rice varieties and their interaction with germination and growth responses under NaCl stress are areas that require further exploration [38]. Additionally, the effect of Spd on photosynthetic pigments, especially under direct seeding and NaCl stress conditions in salt-tolerant rice varieties, is also a phenomenon that has not been fully researched [24,39]. Therefore, this article selected two rice varieties with significantly different salt tolerance levels to investigate the role and effect of Spd in rice of various salt tolerances. This approach allowed for a more comprehensive understanding of the mechanisms by which Spd influenced the response of rice to abiotic stress. Spermidine not only affected the germination characteristics of the rice but also comprehensively affected endogenous hormone levels, antioxidant enzyme activities, osmotic adjustment, and ionic balance in the rice seeds and seedlings. By comparing the two rice varieties, our study revealed the commonalities and differences in Spd under salt stress conditions, providing a new perspective for understanding plant defense mechanisms. This study addressed the important problem of NaCl stress faced by rice under direct seeding conditions in actual agricultural production, making our research highly novel in terms of practical applications.

## 2. Results

### 2.1. Germination Characteristics

**Figure 1:**
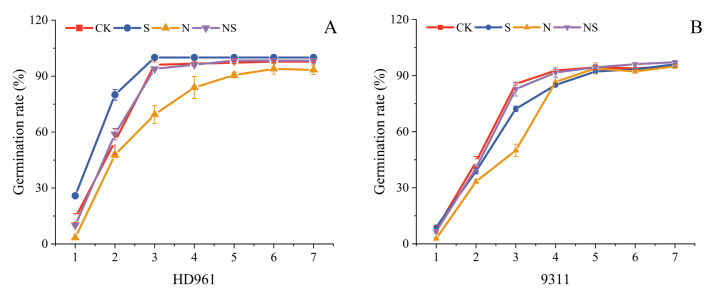
The effect of exogenous spermidine seed soaking on the germination rate of two rice varieties, HD961 (**A**) and 9311 (**B**), under salt stress.

**Figure 2:**
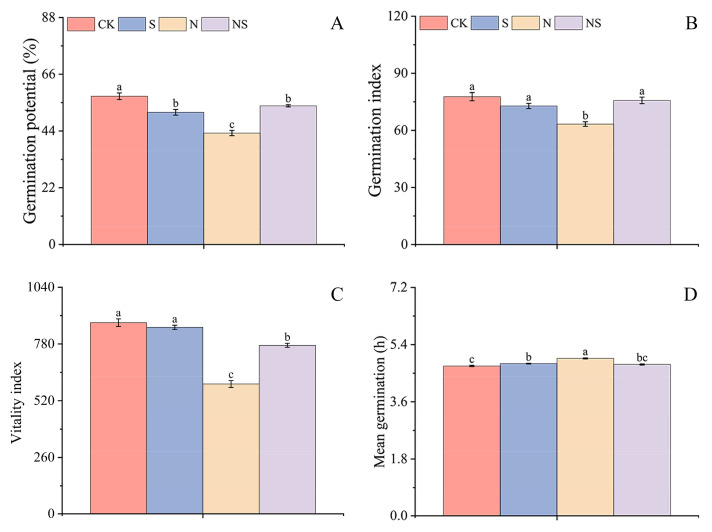
Response mechanism of germination potential (**A**), germination index (**B**), vigor index (**C**), and average germination time (**D**) of 9311 rice seeds to exogenous spermidine under salt stress. Here, S is spermidine seed treatment, N is NaCl treatment, NS is a combination of NaCl and spermidine seed treatments, and CK is no NaCl. In the following figures and tables, S, N, NS, and CK represent the same meanings. Different letters indicate statistically significant differences (*p* < 0.05).

Under salt stress, the soaking of rice seeds with exogenous Spd has a significant effect on germination. The results of this study showed that Spd treatment significantly enhanced those germination characteristics compared to the control (CK) group (Figure 1A,B). In the case of salt-tolerant rice variety HD961 rice seeds, the S treatment increased the germination rate, energy, index, and vigor by 8.61%, 17.51%, and 12.38% under nonstress conditions and by 26.62%, 22.31%, and 34.68% under NaCl stress (Figure S1A–C). Similar improvements were observed for the salt-sensitive rice variety 9311, with increases of 24.45%, 19.67%, and 29.65% under stress conditions (Figure 2A–C). Additionally, it was found that exogenous Spd seed soaking could reduce the average germination time, thereby promoting seed germination in both rice varieties (Figure 2D and Figure S1D). The germination rate and germination potential of the salt-tolerant rice variety HD961 were higher than those of the salt-sensitive rice 9311.

### 2.2. Effects of Spermidine Treatment on the Aboveground and Root Biomass of Rice Seedlings Under NaCl Stress

**Figure 3:**
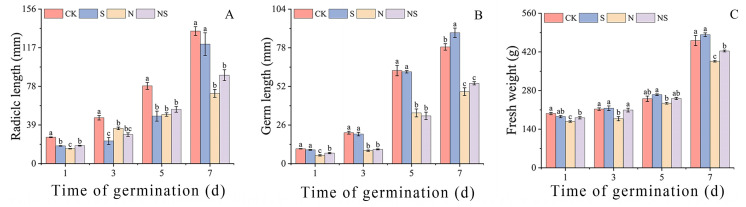
Response mechanism of root length (**A**), shoot length (**B**), and fresh weight (**C**) of 9311 rice variety to exogenous spermidine seed soaking under salt stress for 1, 3, 5, and 7 d. Different letters indicate statistically significant differences (*p* < 0.05).

Exogenous Spd seed soaking improves the biomass of rice germination. Our results showed that, compared to the control conditions, under NaCl stress, the NS treatment significantly promoted the growth of the radicle and plumule of rice seeds. For the HD961 rice variety, on the third day, NS treatment led to a 54.81% increase in radicle length, a 9.68% increase in plumule length, and a 16.81% increase in fresh weight (Figure S2A–C). For the 9311 rice variety, on the fifth day, the NS treatment resulted in increases of 10.59% in radicle length, 0.56% in plumule length, and 7.40% in fresh weight (Figure 3A–C). The effect of exogenous Spd seed soaking on the HD961 rice occurred significantly earlier than that on the salt-sensitive rice variety.

### 2.3. Exogenous Spermidine Treatment Enhanced the Content of Adenosine Triphosphate, Soluble Sugars, and Soluble Starch in Rice Seeds Under NaCl Stress

**Figure 4:**
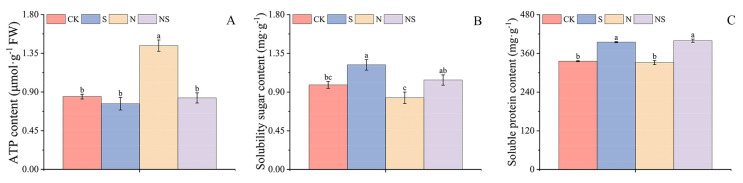
Response mechanism of ATP content (**A**), soluble sugar (**B**), and soluble starch (**C**) in 9311 rice variety to exogenous spermidine seed soaking under NaCl stress. Different letters indicate statistically significant differences (*p* < 0.05).

Exogenous Spd seed soaking affected the adenosine triphosphate (ATP) content and the levels of soluble sugars and proteins in rice seeds under NaCl stress. The results showed that compared to the HD961 variety, the ATP content in the 9311 rice variety increased by 25.93% to 33.36%. In the HD961 variety, the S treatment led to an 8.00% decrease in ATP content compared to CK, and under NaCl stress, the Spd-soaked seed treatment resulted in a 33.27% reduction in ATP content. For the 9311 rice variety, the S and NS treatments, compared to the CK and N treatments, caused a decrease of 9.88% and 42.25%, respectively, in ATP content (Figure 4A and Figure S3A). For both HD961 and 9311 varieties, the S treatment increased the soluble sugar content by 47.36% and 23.72%, respectively, compared to CK. Under NaCl stress, the NS treatment significantly increased the content of soluble sugars and soluble proteins by 22.96%, 24.80%, 5.35%, and 20.47%, respectively (Figure S3B,C). However, there was no significant difference in soluble protein content for the HD961 rice variety. Compared to HD961, the NS treatment in 9311 rice seeds increased the content of soluble sugars and soluble proteins by 1.83% and 15.12%, respectively (Figure 4B,C).

These findings indicate that the developmental capacity of the salt-tolerant rice HD961 is higher than that of salt-sensitive rice varieties. Spd treatment could modulate the metabolic responses of rice seeds to NaCl stress, particularly in terms of energy supply and osmotic adjustment. Spermine exhibited a stronger rescuing ability for the soluble sugar and soluble protein contents in the 9311 rice variety under salt stress. This is crucial for seed germination and stress tolerance.

### 2.4. Effects of Exogenous Spermidine Seed Soaking on Amylase Activity in Rice Seeds Under NaCl Stress

**Figure 5:**
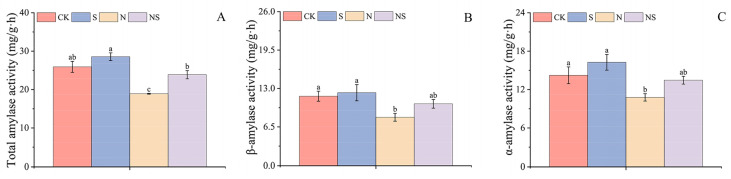
Response mechanisms of total amylase activity (**A**), α-amylase activity (**B**), and β-amylase activity (**C**) in the 9311 rice variety to exogenous spermidine soaking under NaCl stress. Different letters indicate statistically significant differences (*p* < 0.05).

This study also evaluated the effect of NaCl stress on the activity of amylases, which are crucial for seed germination because they hydrolyze starch to provide energy. It was found that NaCl stress inhibited the activity of amylases in the rice seeds. In HD961 rice seeds, the S treatment, compared to CK, significantly increased the activities of total amylase, α-amylase, and β-amylase by 48.57%, 12.12%, and 97.92%, respectively. The NS treatment significantly enhanced those activities by 50.07%, 47.01%, and 3.31% compared to the N treatment. In the 9311 rice variety, although there were no significant differences in α-amylase and β-amylase activities, they increased by 10.14%, 14.32%, 5.04%, 26.26%, 24.93%, and 28.02%, respectively (Figure 5 and Figure S4). The S treatment had a more pronounced promotional effect on the HD961 rice seeds compared to the 9311 rice seeds, indicating a differential response to Spd treatment between the two rice varieties under stress conditions. These results highlighted the role of Spd in modulating amylase activity, which could be a key mechanism by which Spd influences seed germination and energy provision under saline stress.

### 2.5. Exogenous Spermidine Seed Soaking Mitigated the Effect of NaCl Stress on Rice Seed Oxidative Stress

**Figure 6:**
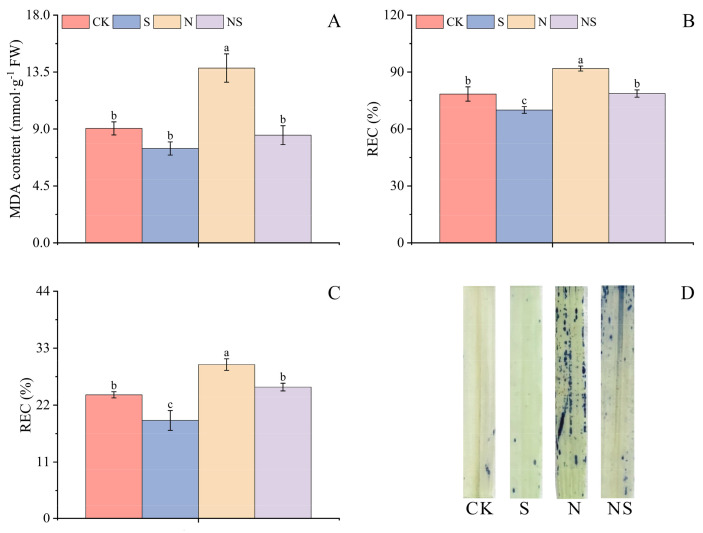
Influence of exogenous spermidine seed soaking on MDA content (**A**), electrolyte leakage in leaves (**B**) and roots (**C**), and H_2_O_2_ distribution in leaves (**D**) of 9311 rice variety under NaCl stress. Different letters indicate statistically significant differences (*p* < 0.05).

This study also examined the effect of NaCl stress on oxidative stress indicators such as MDA and EL in rice seeds. It was observed that Spd seed soaking mitigated oxidative stress by reducing MDA content. Compared to CK, the S treatment in both HD961 and 9311 rice varieties significantly lowered MDA content by 30.54% and 17.50%, respectively. Under NaCl stress, the NS treatment further significantly reduced MDA content by 32.10% and 38.35% in HD961 and 9311, respectively. The 9311 rice seeds showed a higher level of stress than HD961 by 10.35% (Figure 6A and Figure S5A). Notably, in the NS treatment, there was no significant difference in MDA content between the two rice varieties, suggesting that Spd had a more pronounced alleviating effect on the salt-tolerant variety 9311 rice seedlings post-stress. These findings underscored the potential of Spd in modulating the oxidative stress response in rice seeds, particularly in salt-tolerant varieties, and provided valuable insights into the mechanisms by which Spd confers stress tolerance.

Compared to CK, the S treatment in both rice varieties significantly decreased the electrical conductivity of leaves and roots by 39.69%, 26.63%, 10.72%, and 20.60%, respectively. Under NaCl stress, EL increased; however, the NS treatment significantly mitigated that leakage in both rice seedlings’ leaves and roots, with reductions of 7.41%, 20.91%, 14.34%, and 14.56%, respectively. The beneficial effect of Spd seed soaking was more pronounced in the 9311 seedlings variety under NaCl stress than in the HD961 rice seedlings (Figure 6B,C and Figure S5B,C). To further elucidate the effect of Spd seed soaking on rice seedlings under NaCl stress, the leaves were stained with H_2_O_2_, manifested as dark blue spots. In the absence of stress, fewer blue spots were observed in the leaves of CK and S treatments. Under NaCl stress, dense blue spots indicated significant H_2_O_2_ formation in the leaves of rice seedlings with the N treatment, correlating with increased stress intensity. In contrast, the NS treatment significantly reduced H_2_O_2_ accumulation compared to NaCl stress alone (Figure 6D and Figure S5D). These results suggested that Spd seed soaking could protect rice seedlings from oxidative damage induced by NaCl stress, with a more significant protective effect in the salt-sensitive 9311 variety.

### 2.6. Effects of Exogenous Spermidine Seed Soaking on Antioxidant Enzymes and Nonenzymatic Antioxidants in Rice Seeds Under NaCl Stress

**Figure 7:**
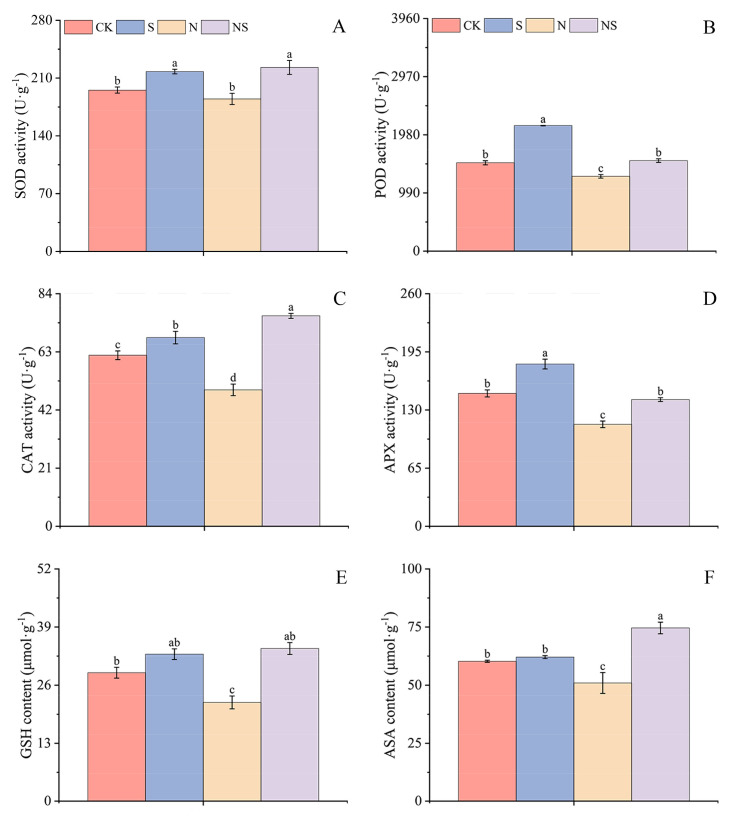
Response mechanisms of superoxide dismutase (**A**), peroxidase (**B**), catalase (**C**), ascorbate peroxidase (**D**), glutathione (**E**), and ascorbic acid (**F**) contents in 9311 rice seeds to exogenous spermidine soaking under NaCl stress. Different letters indicate statistically significant differences (*p* < 0.05).

This study further investigated the effect of Spd seed soaking on the antioxidant enzyme activity and the ascorbic acid (ASA)–glutathione (GSH) cycle in rice seeds under various treatments. It was found that Spd seed soaking promoted the content of antioxidant enzymes and nonenzymatic antioxidants in the rice under NaCl stress. Compared to CK, the S treatment in HD961 rice seeds significantly reduced the activities of superoxide dismutase (SOD), peroxidase, and catalase (CAT) by 6.99%, 21.09%, and 22.04%, respectively. Conversely, in 9311 rice seeds, the activities of those antioxidant enzymes increased by 11.48%, 41.98%, 10.32%, and 22.11%. Under NaCl stress, the activities of antioxidant enzymes in both HD961 and 9311 rice seeds were significantly reduced, with 9311 rice seeds having higher SOD and CAT activities than HD961, whereas peroxidase and ascorbate peroxidase (APX) activities were significantly lower than in HD961. In comparison to NaCl stress alone, the NS treatment significantly increased the activities of SOD, CAT, and APX in HD961 rice seeds by 28.25%, 82.28%, and 30.02%, respectively, and in 9311 rice seeds, by 20.63%, 54.26%, and 24.21% (Figure 7A,F). Additionally, Spd seed soaking significantly increased the GSH and ASA content in the rice seeds under NaCl stress. The activities of GSH and ASA in HD961 and 9311 rice seeds increased significantly by 24.96%, 33.33%, 14.40%, and 2.98% compared to CK, and by 24.93%, 91.34%, 54.39%, and 46.46% compared to NaCl stress alone (Figure S6A–F). These results highlighted the potential of Spd in enhancing the antioxidant defense system of rice seeds under saline conditions, thereby contributing to a better understanding of the mechanisms underlying the stress tolerance conferred by Spd.

### 2.7. Effects of Exogenous Spd Seed Soaking on Hormone Content in Rice Seeds Under NaCl Stress

**Figure 8:**
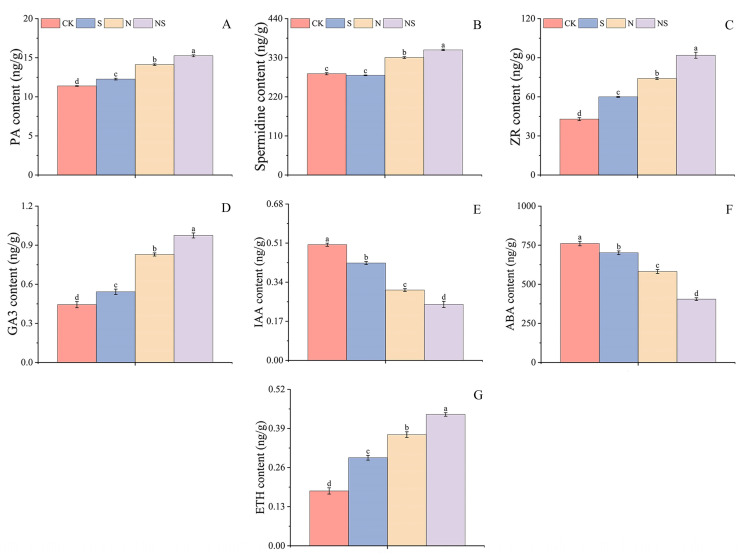
Response mechanism of polyamine content (**A**), spermidine content (**B**), zeatin content (**C**), gibberellin (**D**), auxin (**E**), abscisic acid (**F**), and ethylene content (**G**) in rice seeds to exogenous spermidine soaking under NaCl stress. Different letters indicate statistically significant differences (*p* < 0.05).

Exogenous Spd seed soaking increased the endogenous content of PAs, Spd, and zeatin riboside (ZR) in rice seeds (Figure 8A–C). The results show that compared to CK, the Spd treatment significantly increased the endogenous PA, Spd, and ZR in salt-tolerant rice variety HD961 by 19.83%, 6.94%, and 29.64%, respectively (Figure S7A–C). The Spd treatment in 9311 rice seeds significantly increased the endogenous PA and ZR content by 7.67% and 39.83%, respectively, with no significant difference in endogenous Spd. Under NaCl stress, the salt + Spd (NS) treatment increased endogenous PA by 16.31%, with no significant differences in endogenous Spd and ZR. In 9311 rice seeds, the NS treatment increased PA, Spd, and ZR by 7.99%, 6.46%, and 24.07%, respectively.

Compared to CK, the Spd treatment significantly increased the hormone content in HD961 and 9311 rice seeds: gibberellin 3 (GA3) increased by 9.15% in HD961 and 58.88% in 9311, and ethylene (ETH) increased by 22.19% in HD961 and 60.55% in 9311. Sodium chloride stress significantly reduced the hormone content of GA3 and ETH, but after the NS treatment, GA3 significantly increased by 15.79% and 27.37% in HD961 and 9311, respectively, and ETH significantly increased by 17.61% and 18.16% in HD961 and 9311, respectively. In CK, there were no significant differences in GA3 and ETH hormone content between the HD961 and 9311 rice seeds (Figure S7D–G). In contrast, the content of indole-3-acetic acid (IAA) and ABA hormones, compared to CK, was significantly reduced by the Spd treatment by 14.91%, 11.84%, 15.85%, and 7.77%. The ABA content in the HD961 rice seeds showed no significant difference under NaCl stress with the NS treatment, whereas the IAA hormone content significantly decreased by 18.01%, and in 9311 rice seeds, it significantly decreased by 20.44% and 24.08% (Figure 8D–G).

### 2.8. Effects of Exogenous Spermidine Seed Soaking on Morphological Indicators of Rice Seedlings Under NaCl Stress

Compared to CK, the Spd treatment significantly influenced the morphological indicators of HD961 and 9311 rice seedlings. As shown in Table 1, for HD961 rice seeds, the S treatment led to a 10.89% increase in seedling height, a 24.69% increase in basal stem width, and a 23.85% increase in aboveground fresh weight. In contrast, for 9311 rice seeds, the S treatment resulted in a 63.80% increase in root length, a 23.71% increase in basal stem width, and an 11.31% increase in leaf area, as well as increases in aboveground and belowground fresh weight and dry weight of 27.14%, 31.48%, 48.61%, and 37.72%, respectively. The Spd seed soaking had a more pronounced effect on enhancing morphological indicators in 9311 rice seedlings compared to HD961. Under NaCl stress, the NS treatment significantly promoted the growth of various morphological indicators in 9311 rice seedlings, with increases of 48.75% in plant height and 37.41% in root length. Compared with the N treatment, the NS treatment resulted in a leaf area that was 53.84% greater and a stem-base width that was 104.29% greater than those observed in the N treatment. The differential response to Spd seed soaking under NaCl stress was particularly notable in the salt-sensitive 9311 rice seedlings, with the NS treatment leading to a significant increase in belowground biomass (Figure S8). These results highlighted the role of Spd in mitigating the adverse effects of NaCl stress on rice seedling growth, especially in salt-sensitive varieties like 9311.

### 2.9. Effects of Exogenous Spermidine Seed Soaking on Photosynthetic Pigment Content in Rice Seedlings Under NaCl Stress

**Figure 9:**
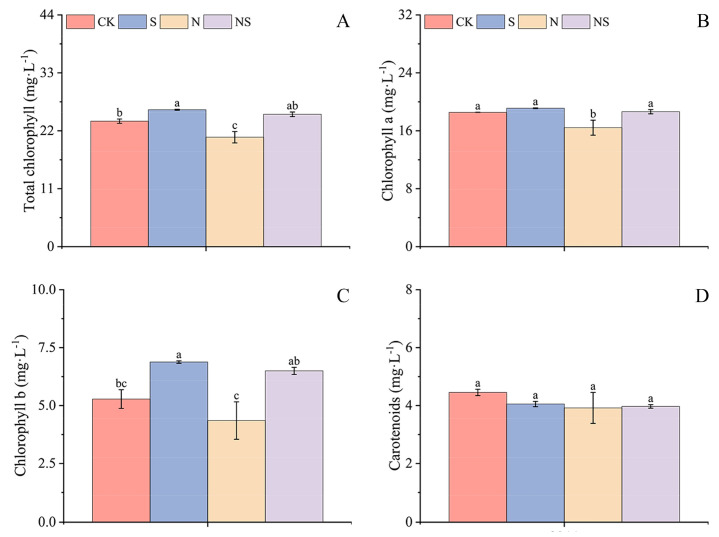
Response mechanism to exogenous spermidine soaking under NaCl stress of total chlorophyll (**A**), chlorophyll a (**B**), chlorophyll b (**C**), and carotenoid (**D**) contents of 9311 rice seedlings. Different letters indicate statistically significant differences (*p* < 0.05).

Compared to CK, exogenous Spd seed priming significantly enhanced the chlorophyll content in rice seedlings. In HD961 rice seedlings, the S treatment significantly increased the content of chlorophyll a, chlorophyll b, and total chlorophyll by 9.88%, 8.51%, and 9.51%, respectively, with no significant change in carotenoid content (Figure 9D and Figure S9D). Similarly, in 9311 rice seedlings, the S treatment increased the content of chlorophyll b and total chlorophyll by 30.21% and 9.07%, respectively (Figure 9A–C). Under NaCl stress, the chlorophyll content in rice seedlings was significantly reduced. However, after the NS treatment, the content of chlorophyll a, chlorophyll b, and total chlorophyll in both HD961 and 9311 rice seedlings significantly increased compared to NaCl stress by 40.69%, 39.68%, 40.47%, 13.44%, 49.22%, and 20.94%, respectively. Among those, HD961 rice seedlings showed the most significant content of chlorophyll a under NaCl stress, and 9311 rice seedlings had the highest increase in chlorophyll b content (Figure S9A–C). Under salt stress, spermine more significantly promoted the chlorophyll content of the salt-sensitive rice 9311.

**Figure 10:**
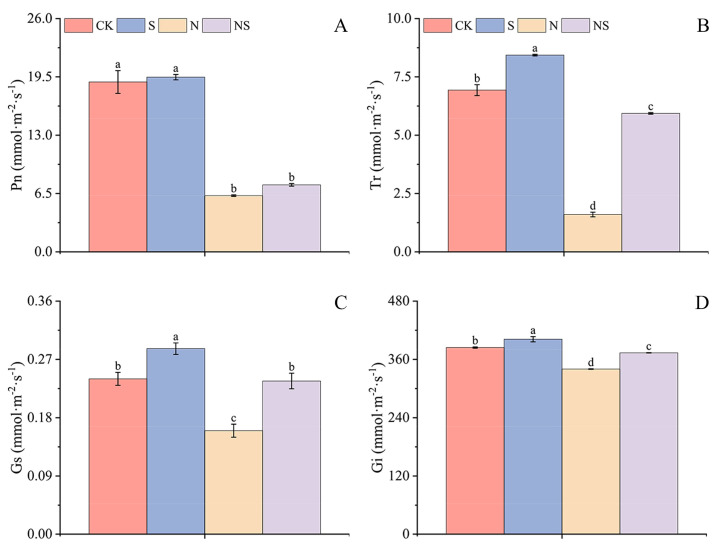
Response mechanism to exogenous spermidine soaking under NaCl stress of net photosynthetic rate (**A**), transpiration rate (**B**), stomatal conductance (**C**), and intercellular carbon dioxide concentration (**D**) of 9311 rice seedlings. Different letters indicate statistically significant differences (*p* < 0.05).

Compared to CK, the S treatment significantly increased the photosynthetic rate, stomatal conductance, internal CO_2_ concentration, and transpiration rate in HD961 and 9311 rice seedlings by 12.01%, 58.69%, 4.84%, and 29.05%, respectively, for HD961, and by 2.81%, 19.44%, 4.47%, and 21.63%, respectively, for 9311. Sodium chloride stress significantly reduced those photosynthetic parameters in the rice seedlings by 23.55%, 32.60%, 26.23%, and 10.61%, respectively, for HD961, and by 66.90%, 33.33%, 11.54%, and 76.92%, respectively, for 9311, indicating that 9311 rice seedlings suffered greater damage to photosynthetic indicators under NaCl stress than the HD961 rice seedlings. However, after the NS treatment, compared to NaCl stress, the photosynthetic rate, internal CO_2_ concentration, and transpiration rate in HD961 rice seedlings significantly increased by 16.35%, 5.17%, and 13.12%, respectively, and in 9311 rice seedlings, stomatal conductance, internal CO_2_ concentration, and transpiration rate significantly increased by 47.91%, 9.87%, and 270.83%, respectively (Figure 10 and Figure S10).

### 2.10. Effects of Exogenous Spermidine Seed Soaking on Ion Content in Rice Seedlings Under NaCl Stress

As shown in Table 2 and Table S1, compared to CK, the Spd treatment increased the content of Na^+^, Cl^−^, K^+^, and Ca^2+^ in HD961 rice seedling leaves by 105.87%, 110.01%, 12.31%, and 23.69%, respectively, and decreased the content of Na^+^, Cl^−^, and K^+^ in the root system by 26.20%, 15.67%, and 5.05%, respectively. In the leaves and roots of HD961 rice seedlings, the NS treatment increased the content of Na^+^ and Cl^−^ while decreasing K^+^ and Ca^2+^. Compared to the N treatment, NS significantly reduced the content of Na^+^ and Cl^−^ in rice leaves and roots by 20.94%, 49.50%, 35.58%, and 6.21%, respectively, and increased the content of K^+^ and Ca^2+^ by 91.98%, 33.61%, 33.70%, and 33.32%, respectively. Compared to CK, the Spd treatment significantly reduced the content of Na^+^ and Cl^−^ in 9311 rice seedling leaves and roots by 40.38%, 33.96%, 33.64%, and 40.92%, and increased the Ca^2+^ content by 13.99% and 38.78%. The N treatment significantly increased Na^+^ and Cl^−^ by 98.52%, 107.36%, 359.04%, and 59.62%, respectively. Compared to NaCl stress, the NS treatment significantly reduced the content of Na^+^ and Cl^−^ in rice seedling leaves and roots by 47.43%, 43.00%, 14.76%, and 37.79%, respectively, and increased the content of K^+^ and Ca^2+^ by 66.22%, 37.69%, 8.56%, and 137.36%, respectively. Under NaCl stress, Na^+^ significantly increased in the leaves of HD961 rice seedlings and the roots of 9311 rice seedlings. In the NS treatment, K^+^ content in the leaves of HD961 rice seedlings and Ca^2+^ content in the roots of 9311 rice seedlings showed significant increases.

**Figure 11:**
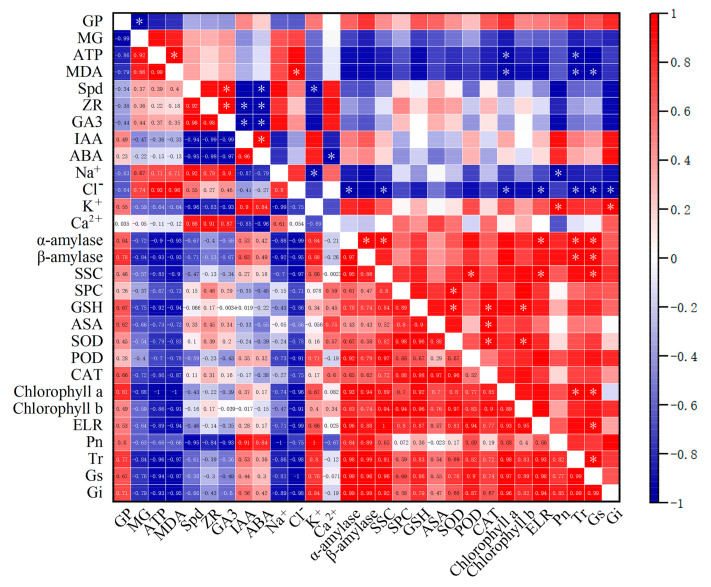
Response mechanism of rice seed germination and seedling growth to exogenous spermidine on HD961 and 9311 under salt stress. Red indicates a positive correlation between the two parameters, and blue indicates a negative correlation. * *p* ≤ 0.05.

By analyzing the correlations between rice seed germination and physiological and photosynthetic processes, a correlation matrix was constructed to compare the correlations among various indices (Figure 11 and Figure S11). Based on the correlation analysis results, we could conclude that for the two rice varieties, there was a positive correlation between antioxidant enzymes, photosynthetic pigments, and amylases, whereas a negative correlation was observed with ATP, MDA, and endogenous hormones. Notably, the salt-sensitive rice variety 9311 was more significantly affected by stomatal conductance. Figure 11 shows that the salt-sensitive variety 9311 had more indices significantly positively affected, whereas the salt-tolerant variety HD961 had relatively fewer indices significantly affected.

## 3. Discussion

In the plant growth process, seed germination marks the first step of the life cycle, and the seedling stage is crucial for the entire life cycle, with these two stages being most susceptible to external environmental influences. Among these, NaCl stress is one of the leading environmental factors limiting rice growth [40]. Seed soaking treatment is an economical and effective method that can reduce costs and improve the seedling establishment rate of rice [20]. Research indicates that an appropriate concentration of Spd can enhance the germination rate of seeds such as cucumber and rice [41], promote the growth and development of crops, significantly affect the growth of secondary roots in rice seedlings, and improve the quality of seeds at the maturation stage of rice [42]. In experiments, Spd seed soaking has alleviated the decline in rice biomass under NaCl stress, mitigated ionic toxicity by increasing the activity of antioxidant enzymes, promoted starch hydrolysis, and enhanced the activity of seed amylase. Furthermore, Spd can balance the endogenous hormones in rice seedlings under NaCl stress and improve chlorophyll’s photosynthetic capacity, thereby alleviating salt stress’s effect on rice’s germination and seedling growth.

### 3.1. Exogenous Spermidine Promotes Seed Germination and Seedling Growth by Alleviating Ion Toxicity and Protecting Membranes from Damage

Research indicates that NaCl stress leads to the accumulation of Na+ and Cl^−^ in the stems of plants, which in turn triggers the loss of K^+^, Ca^2+^, and Mg^2+^. This ion imbalance not only hinders the elongation of plant stems and the production of new leaves but also compromises the integrity of cell membranes and affects cellular structure [10,42]. Within the plant, Ca^2+^ plays a pivotal role in the signal transduction that detects environmental changes and leads to plant adaptive reactions; regulating ion balance is crucial for rice seedling growth [13]. The intervention of exogenous substances can reduce the translocation of Na+ from the underground to the aboveground parts. This study showed that under NaCl stress, Na^+^ and Cl^−^ contents in both rice varieties increased, with the ion content in the underground parts significantly higher than in the aboveground parts. The salt-sensitive rice variety 9311 had higher Na^+^ and Cl^−^ ion contents than the salt-tolerant variety HD961. After the application of Spd, K^+^ and Ca^2+^ returned to a relatively stable state, indicating that NaCl stress inflicted more severe damage on salt-sensitive rice. Soaking seeds in Spd reduced the Na+ and Cl^−^ content in both rice varieties, alleviating the stress-induced damage to rice, suggesting that Spd stabilized the ion balance by mitigating ion toxicity, thereby promoting the germination [43]. This outcome was similar to the responses of crops such as fescue, wheat, and corn under adverse stress conditions [44]. Sodium chloride stress can disrupt the stability of cell membranes and, by interfering with the absorption of water and nutrients, affect the growth of plants such as rice and sorghum and accelerate rice leaves’ senescence [45]. Sodium chloride stress can also inhibit the growth of the primary root system and the development of lateral roots, leading to a reduction in biomass [46]. This study found that salt stress significantly reduces the biomass of rice, inhibits the growth of rice, and accelerates the withering speed of rice leaves. Research has found that applying exogenous regulators can effectively promote seed germination under NaCl stress and improve the root growth of rice seeds [40]. In this study, NaCl stress reduced the germination rate of the two rice varieties, prolonged the average germination duration, decreased the biomass of both the aboveground and belowground parts of rice seedlings, and reduced rice seedlings’ fresh and dry weights. The germination rate of salt-tolerant rice was higher, but the alleviating effect of spermine on salt-sensitive rice was stronger. This indicated that exogenous Spd can mitigate the morphological damage to rice caused by NaCl stress. Different varieties have varying resistances to stress; however, studies have shown that Spd seed soaking can increase the dry matter content of both rice varieties. Under NaCl stress, Spd had a more pronounced inhibitory effect on the germination and seedling growth of the salt-sensitive rice variety 9311. This was similar to the research results of Du [47], which indicate that the regulatory effect of exogenous plant growth regulators on salt-sensitive rice varieties is greater than that on salt-tolerant rice varieties.

### 3.2. Exogenous Spermidine Modulates Amylase Activity to Promote Starch Hydrolysis, Providing Energy for Seed Germination

Starch is the end product of photosynthesis in plants [18], serving not only as the primary storage form of carbohydrates in seeds but also as one of the plants’ energy sources [48]. During seed germination, starch degradation yields metabolites. When plants are subjected to NaCl stress, they modulate starch metabolic pathways to increase sugar accumulation, thereby enhancing their salt tolerance. Soluble sugars can regulate the production of ROS in plants under stress [49]. Research has found that treatment with exogenous regulators reduces the content of starch and ATP in seeds while increasing the activity of amylase and the content of soluble sugars. By regulating the accumulation of total sugars [50], exogenous regulators enhance photosynthesis in chloroplasts and the accumulation of carbohydrates, thereby increasing the content of soluble sugars in leaves [51]. This study found that under NaCl stress, the content of soluble sugars and soluble proteins in the two rice varieties decreased, indicating that NaCl stress inhibited the conversion of starch into sugars, reducing the energy produced during the germination of rice seeds. After treatment with Spd, the contents of soluble sugars and soluble proteins in both rice varieties were significantly increased, particularly in the salt-sensitive rice variety 9311. This might have been the result of exogenous Spd alleviating the rate of starch degradation under NaCl stress, a finding that has been confirmed in studies on wheat germination [25]. During the germination process of quinoa seeds, α-amylase facilitates the hydrolysis of starch. As the seeds continuously absorb water, stored compounds are degraded by the action of hydrolytic enzymes [52], providing the seeds with essential nutrients. Studies have also found that applying Spd during the grain-filling stage significantly increases the content of α-amylase, β-amylase, fructose, and sucrose, enhancing the activity of starch by increasing the amylose content. However, the content of amylases in quinoa seeds continuously declines throughout the germination period, possibly due to the conversion of the amylases into glucose and other substances during germination [42]. This study found that the total starch content significantly decreased under NaCl stress. The salt-sensitive rice variety 9311 has a higher starch content than the salt-tolerant variety HD961, which might be because the former’s seeds are more filled. After applying exogenous Spd, the α-amylase activity in the salt-sensitive rice variety 9311 was lower than that in the salt-tolerant variety HD961, whereas the β-amylase activity was the opposite, with the 9311 variety having a higher content. This suggested that the salt-tolerant rice variety HD961 might enhance the total amylase activity by increasing α-amylase activity, whereas the 9311 variety increased the total amylase activity by enhancing β-amylase activity. Applying Spd can improve the activity of wheat seeds under high temperatures [41], and both seed soaking and foliar spraying can ensure a continuous supply of ATP [26]. This study found that under NaCl stress conditions, the salt-sensitive rice variety 9311 had the highest ATP content, indicating that ion toxicity and osmotic stress severely limited the release of energy during the germination process of rice seeds under salt stress [25]. After treatment with Spd, the ATP content of the salt-tolerant rice variety HD961 was significantly lower than that of the 9311 variety. Exogenous Spd promotes the metabolism of energy substances during seed germination, thereby accelerating germination [53]. The higher activity of ATPases might be related to the absorption and protection of nutrients by Spd and salicylic acid (SA) [26]. Exogenous Spd enhances glycolysis and mitochondrial function, improving the germination capacity of seeds and increasing the respiratory rate and ATP levels in rice seeds and seedlings [54].

### 3.3. Exogenous Spermidine Mitigates NaCl Stress in Rice Seed Germination by Regulating Antioxidant Enzymes and Nonenzymatic Antioxidants

Under abiotic stress conditions, oxidative stress inevitably leads to the generation of MDA [21], triggering increased H_2_O_2_ levels and continuous ROS accumulation, causing cellular membrane damage. Antioxidant enzymes and nonenzymatic antioxidants are widely distributed in cells [55], effectively modulating ROS balance and scavenging oxygen radicals [56]. Superoxide dismutase reduces oxygen radical content, decreasing toxic hydroxyl radical formation, and protects plants from ion toxicity induced by abiotic stress. Glutathione and ASA, through the ASA–GSH cycle, enhance APX and CAT activities, reducing H_2_O_2_ production [26]. Studies indicate that exogenous melatonin can promote the antioxidant system and boost starch metabolism [57], improving corn seed germination and growth. Exogenous Spd maintains chlorophyll function and photosynthesis, preserves cellular integrity, increases antioxidant enzyme activity in tomato seedlings, alleviates oxidative damage, clears ROS from leaves, and promotes plant seedling growth [58]. This study found that under NaCl stress, the salt-sensitive rice variety 9311 had higher levels of MDA, relative electrical conductivity, and H_2_O_2_ compared to the salt-tolerant variety HD961, with less EL in the underground parts than in the aboveground parts of rice seedlings. Hydrogen peroxide staining experiments on leaves further confirmed that NaCl stress led to many dark blue spots, indicating more severe cellular damage in the salt-sensitive rice. Additionally, the translocation of salts from the roots to the aboveground parts was influenced by the exogenous application of Spd, which significantly enhanced the antioxidant enzyme activity in rice seeds, suggesting that Spd might induce endogenous hormones to boost that activity, protect cell membranes, and reduce damage from abiotic stress. The ASA–GSH cycle is an important antioxidant mechanism within plants, activating seed antioxidant enzyme activity; ASA and GSH, as small-molecule antioxidants [59], can directly scavenge ROS and provide overall detoxification. It was also found that foliar application of protospacer adjacent motif promoted the ASA–GSH cycle, reducing internal H_2_O_2_ levels in seeds [56] and maintaining plant redox balance. The application of PA is closely related to plant tolerance to abiotic stress. Because PA can eliminate oxygen-free radicals and their accumulation, it helps maintain ROS homeostasis within cells [13]. This study found that SA and spermine can reduce ROS accumulation and enhance plants’ photosynthetic capacity under NaCl stress, thereby promoting plant growth. Seed priming agents generally positively affect plant enzyme activity [60], with significant differences in effects depending on the priming agent used. Our results showed that in the two rice varieties, the antioxidant enzyme activity in HD961 was higher than that in the salt-sensitive rice 9311, and the enzyme activity of ASA and GSH was positively correlated with antioxidant enzyme activity. Under NaCl stress, the efficiency of the ASA–GSH cycle was enhanced, and the content of MDA and the amount of EL were reduced. These findings confirmed that exogenous Spd could increase the antioxidant enzyme activity in seeds and enhance the efficiency of the ASA–GSH cycle, thereby improving seed germination under abiotic stress and promoting seedling growth. This result was consistent with studies showing that antioxidant enzymes clear ROS in aged oat seeds, promoting seed germination [61].

### 3.4. Exogenous Spermidine Promotes Rice Seed Germination by Regulating Endogenous Hormones

Endogenous hormones are crucial in regulating plant growth [62], with seed germination capacity depending primarily on the balance between ABA and GA [27]. These two plant hormones reciprocally regulate each other’s metabolism [4]. Changes in the external environment can affect the homeostasis of plant endogenous hormones. Reactive oxygen species are involved in seed germination and seedling growth [63], inhibiting seed germination by affecting ABA and GA. Gibberellin content is closely related to the biosynthesis of α-amylase [52], and ZR promotes photosynthesis and delays cellular damage by enhancing the integrity of cell membranes [64]. Studies show that PAs can be catabolized to Spd and further form putrescine [65]. Under drought stress, hormones regulate the activity of PAs, which is essential for normal plant growth and development. In terms of the nutritional quality of rice, Spd maintains internal hormonal balance by enhancing PA and reducing ETH production [66], and melatonin promotes seed germination by decreasing ABA content. Indole-3-acetic acid, jasmonic acid, and ABA act together to promote seed germination and enhance plant growth capacity [18]. This study found that under NaCl stress, PA, Spd, and ZR significantly increased during the germination process of rice seeds, whereas the Spd content in the salt-tolerant rice HD961 did not rise significantly. This indicated that exogenous Spd application could increase endogenous PA content, which was consistent with research showing that exogenous Spd modulates endogenous hormones to alleviate damage to corn seedlings [64]. Exogenous Spd seed soaking reduced the levels of IAA and ABA in both rice seed types under NaCl stress while increasing the levels of GA3 and ETH, and the content of GA3 in the salt-tolerant HD961 rice was higher compared to that in the salt-sensitive 9311 rice, suggesting that exogenous Spd promoted seed germination by increasing PA and auxin content. The decrease in ETH levels might have been due to higher PA accumulation inhibiting the conversion of 1-aminocyclopropane-1-carboxylic acid to ethylene [67]. The ABA content in the salt-tolerant rice HD961 did not significantly decrease, possibly because endogenous GA1 in the seeds was not detected, which did not affect the levels of endogenous ABA and GA4 [59]. Brassinosteroid seed soaking can regulate the levels of endogenous hormones in plants [62], and in rye, it reduces ABA content under stress conditions, with GA3 stimulating germination by modulating cell-wall-related transcription factors [27], maintaining the normal physiological and biochemical functions of plants.

### 3.5. Exogenous Spermidine Enhances Chlorophyll Pigments in Rice Seedlings, Thereby Promoting Seedling Growth

This study found that chlorophyll and photosynthesis not only directly affected plant growth conditions but were also key physiological indicators of plant salt tolerance [62]. Gas exchange is considered a good indicator for assessing plant growth conditions, and the stability of chlorophyll is conducive to maintaining normal photosynthesis [43]. Sodium chloride stress increases water loss, reduces leaf photosynthetic efficiency, and accelerates chlorophyll’s breakdown, disrupting the chloroplast ultrastructure [56], leading to a decrease in plant biomass. A decrease in soluble sugar content might lead to excessive sucrose consumption by leaves, inhibiting the growth of aboveground parts [68]. Gibberellin affects the activity of photosynthetic enzymes, thereby reducing the efficiency of plant photosynthesis [67]. Studies have shown that exogenous application of Spd can maintain water in the leaves of tomato seedlings, reduce transpiration rates, and improve gas exchange and salt tolerance [43]. Foliar application of regulators can reduce oxidative damage and maintain the content of chloroplasts and the stability of cell membranes under cadmium stress [56,68]. This study found that the chlorophyll content in rice seedlings under NaCl stress significantly decreased. The salt-tolerant rice variety HD961 reduced chlorophyll a content, whereas the salt-sensitive variety 9311 reduced chlorophyll b content, leading to a decrease in total chlorophyll content. That might have been why the ATP content in the salt-sensitive variety 9311 was higher than in the salt-tolerant variety HD961, affecting the absorption of light waves by the two different rice varieties under stress. After exogenous Spd treatment, the chlorophyll content significantly rebounded, possibly due to enhanced photosynthesis, which slowed the degradation of chlorophyll and increased its synthetic capacity. The photosynthetic pigments in chloroplasts closely affect the efficiency of photosynthesis, and a decrease in chlorophyll content can adversely affect gas exchange in leaves [69]. The experimental results indicated that under NaCl stress, the net photosynthetic rate, stomatal conductance, intracellular carbon dioxide concentration, and transpiration rate of rice leaves were all significantly reduced. The stomatal conductance of the salt-sensitive rice variety 9311 was inhibited to a greater extent than that of the salt-tolerant variety HD961, possibly because the salt-sensitive rice primarily suppressed photosynthesis by increasing water evaporation and disrupting the stomatal exchange pathway with the external environment under NaCl stress. Exogenous Spd can regulate photosynthetic pigments and stomatal conductance, maintain ionic balance, and protect cell membranes under NaCl stress, promoting the germination of rice seeds and the accumulation of seedling biomass, which is consistent with the findings that Spd enhances the heat tolerance of tomato seedlings by strengthening photosynthesis and cellular redox homeostasis [70].

## 4. Materials and Methods

### 4.1. Experimental Materials

Rice materials: HD961 is a salt-tolerant local rice variety planted in coastal areas; it is disease-resistant, insect-resistant, and salt-tolerant, and exhibits vigorous growth [71]. A previous study demonstrated that the 9311 variety was significantly reduced at a NaCl concentration of 0.5%, showing high salt stress sensitivity; thus, it is an ideal material for studying differences in salt tolerance [72]. Feng et al. reported that there are significant differences in growth, antioxidant capacity, and yield between HD961 and 9311 under salt stress [47]. Compared with 9311, HD961 showed better growth and antioxidant capacity under salt stress. In a study evaluating the physiological response and salt tolerance of different rice varieties to NaCl stress, HD961 and 9311 showed different degrees of salt tolerance, providing a basis for comparison. The salt-tolerant biotype rice variety HD961 and the salt-sensitive biotype rice variety 9311 were provided by the seed resource bank of Binhai Agricultural College of Guangdong Ocean University.

Spermidine (AR) was purchased from Beijing Suolaibao Technology (Beijing, China).

### 4.2. Experimental Design

The experiments were conducted from September 2022 to October 2024 at the South China Center of the National Center for Salt-Tolerant Rice Technology Innovation, Guangdong Ocean University. Fully developed, uniformly sized rice seeds free from mold and pests were selected. The seeds were disinfected with 3% H_2_O_2_ for 10 min, then thoroughly rinsed with deionized water until no H_2_O_2_ residue remained. The germination test was conducted in accordance with international seed testing procedures using a filter-paper bed. Two layers of qualitative filter paper were laid at the bottom of a germination box (12.0 cm × 12.0 cm × 5.4 cm), and 15 mL of distilled water or 100 mmol·L^−1^ NaCl solution was added. One hundred test seeds were placed on the filter paper and incubated in a smart light incubator (Ningbo Safe Test Instruments Co., Ltd. PGX-450D, Ningbo, China) at a temperature of 30 °C in the dark for 24 h; they were then transferred to a smart large-scale artificial climate chamber (Guangzhou Jidi Instruments JIDI-PX1100, Guangzhou, China) for a 7 d cultivation period under the following conditions: a photoperiod of 12 h/d, day/night temperatures of 28 °C/23 °C, light intensity of 15,000 lx, and relative humidity of 70%. After 1, 3, 5, and 7 d of germination, 5 random seedlings from each treatment were selected to measure the lengths of the radicle and plumule using a vernier caliper (accuracy of 0.01 mm) and the fresh weight of 5 whole seedlings was determined using an analytical balance (accuracy of 0.001 mg). At 48 h post-germination, a random sample of germinating seeds was taken and stored at −80 °C for subsequent analysis.

The seedling growth experiment was conducted in the greenhouse of the Binhai Agricultural Science Institute, Guangdong Ocean University, Zhanjiang City, Guangdong Province. Fully developed and intact seeds were selected and disinfected with a 3% H_2_O_2_ solution for 10 min, followed by thorough rinsing with distilled water until no residue remained. The seeds were then primed with Spd at a 0.1 mmol·L^−1^ concentration and germinated in the dark at 30 °C for 48 h. Subsequently, 75 uniform seeds were sown into plastic pots (with an upper diameter of 19 cm, lower diameter of 14 cm, and height of 17 cm, lined with 2 layers of punched paper at the bottom), each filled with 3 kg of test soil (a volumetric ratio of laterite to sand of 3:1) [69]. When the rice seedlings reached the 4-leaf-1-tiller stage (approximately 30 d old), morphological indicators were measured, and their leaves and roots were harvested, flash-frozen in liquid nitrogen, and stored at −80 °C for subsequent analysis.

The experiment was set up with 4 treatments: CK with deionized water, Spd treatment with 0.1 mmol·L^−1^, salt treatment with 100 mmol·L^−1^ NaCl, and salt + Spd treatment with 100 mmol·L^−1^ NaCl + 0.1 mmol·L^−1^.

### 4.3. Measurement Indices and Methods

#### 4.3.1. Measurement of Seed Germination Parameters

Rice germination was considered to have occurred when the length of the plumule exceeded half the length of the seed itself [48]. The number of germinated seeds was surveyed daily, and on the seventh day, the germination percentage, germination index, and vigor index were calculated. The formulas for these calculations were as follows:

Germination percentage = number of germinated seeds/total number of seeds

Germination energy = number of seeds germinated in the first 3 d/total number of seeds

Germination index (GI) = Σ (Gt/Dt)

Vigour Index = S × GI

In the formulas, S is the length of the plumule, calculated as the average length after 1 week of germination under different treatments; Gt is the number of seeds germinated on day t; and Dt is the number of days for seed germination.

#### 4.3.2. Determination of Malondialdehyde, Soluble Sugar, and Soluble Protein Content

For rice germination, first, 0.5 g of rice seeds was ground and mixed with 10 mL of 10% trichloroacetic acid to form a homogenate and then centrifuged (4 °C, 6000 rpm, 20 min). The content of MDA was determined using the thiobarbituric acid method [18], with absorbance measurements taken at 450, 532, and 600 nm. A total of 1 g of rice seeds was ground and mixed with 0.8 mL of 80% ethanol to form a homogenate, then transferred to a capped centrifuge tube, brought up to volume with 80% ethanol to 10 mL, placed on ice, and centrifuged (4 °C, 10,000 rpm, 20 min). The supernatant was reserved for the determination of soluble sugars using a biochemical parameter test kit (Suzhou Greis Biotechnology, G0857W, Suzhou, China). We took 0.5 g of rice seed samples and placed it in a precooled mortar, added 10 mL of 0.05 mol precooled phosphate buffer (pH 7.8) in 3 portions, ground it on ice to form a homogenate, transferred it to a centrifuge tube, and centrifuged it at 4 °C, 10,000 rpm, for 20 min. The supernatant was a crude protein extract. To 1 mL of enzyme solution, 5 mL of Coomassie brilliant blue solution was added. The solution was mixed well, and after a 2 min reaction, the absorbance was measured at 595 nm [73].

#### 4.3.3. Determination of Adenosine Triphosphate Content

Adenosine triphosphate was measured using an ATP assay kit (Suzhou Greis Biotechnology Co., Ltd. G0857W, Suzhou, China) to determine ATP content [26].

#### 4.3.4. Determination of Amylase Activity

The following procedure was used to determine amylase activity: Weigh 1 g of rice seeds and place it in a mortar with 2 mL of pH 5.6 citrate buffer and a small amount of quartz sand. Grind into a slurry, then transfer to a centrifuge tube. Rinse the mortar with 8 mL of pH 5.6 citrate buffer and transfer the residue to a 50 mL volumetric flask. Use distilled water at 40 °C to rinse the mortar, then extract in a water bath at 40 °C for 1 h, stirring occasionally to ensure complete extraction. After the extraction is complete and the mixture has cooled to room temperature, pour the extract into a 10 mL centrifuge tube and centrifuge at 3000 rpm for 10 min. The supernatant was the amylase extract, which was used to measure amylase activity. The 3,5-dinitrosalicylic acid colorimetric method was used for that determination [25].

#### 4.3.5. Determination of Antioxidant Enzymes, Reduced Glutathione, and Reduced Ascorbic Acid Content

The determination of SOD activity was performed using the nitro blue tetrazolium photochemical reduction method, where the activity of peroxidase was measured using the guaiacol method [51]. The activities of CAT and APX were determined using the UV spectrophotometry method [74]. The content of AsA was measured using the 4,7-diphenyl-1,10-phenanthroline colorimetric method, and the content of GSH was measured using the 5,5′-dithiobis-2-nitrobenzoic acid colorimetric method [75].

#### 4.3.6. Determination of Endogenous Hormone Content

Endogenous plant hormones Spd, putrescine, GA3, ETH, ABA, ZR, and IAA were measured using Shanghai enzyme-linked ELISA detection kits. The absorbance [optical density (OD) value] at a 450 nm wavelength was measured using a microplate reader, and sample concentrations were calculated.

#### 4.3.7. Determination of Seedling Growth Parameters

The following procedure was used to determine the seedling growth parameters: When the seedlings reach the 4-leaf-1-heart stage, rinse the rice seedlings from each treatment with tap water, blot dry with absorbent paper, and separate the aboveground and belowground parts for morphological measurements. Select 20 representative rice seedlings from each treatment. Measure the plant height and basal stem width of each individual plant using a ruler and calipers. Weigh the fresh weight of the aboveground and belowground parts with an electronic balance. Then, dry the seedlings at 105 °C for 30 min and then at 80 °C until a constant weight is reached to determine the dry weight of the aboveground parts and the root system [69].

#### 4.3.8. Measurement of Electrical Conductivity

Relative electrical conductivity was measured using a conductivity meter (DDSJ-308F, Lei Ci, Shanghai, China). We chopped 0.5 g of leaves and roots, placed it in 10 mL of distilled water, soaked it for 4 h, shook it well, and measured the initial electrical conductivity (EC1). The samples were boiled in a water bath for 10 min and cooled to room temperature; then, the final electrical conductivity (EC2) was measured. The conductivity was assessed as relative electrical conductivity (%) = (EC1/EC2) × 100% [10].

#### 4.3.9. Determination of Chlorophyll Content

The determination of chlorophyll content in the leaf tissues was performed using the ethanol extraction method, as follows: Cut the seedling leaves with scissors, weigh 0.1 g, and place it in a 10 mL centrifuge tube. Add 10 mL of anhydrous ethanol for extraction. Keep the test tubes in the dark for 24 h. Record the OD values of the extract at 665, 649, and 470 nm wavelengths using a UV-Vis spectrophotometer. Calculate the content of chlorophyll a (Chl a), chlorophyll b (Chl b), total chlorophyll (Total Chl), and carotenoids (Car) using the following formulas [76]:

Chl a (mg g^−1^) = 13.95 × OD 665 − 6.88 × OD 649

Chl b (mg g^−1^) = 24.96 × OD 649 − 7.32 × OD 665

Total Chl (mg g^−1^) = Chl a + Chl b

Car (mg g^−1^) = (1000 × OD 470 − 2.05 × Chl a − 114.8 × Chl b)/245

#### 4.3.10. Determination of Ion Content

The determination was performed using Shanghai enzyme-linked ELISA detection kits, and the ions Na⁻, Cl⁻, K⁺, and Ca²⁺ were detected using an inductively coupled plasma mass spectrometer (ICP-MS, Agilent 7700, Agilent Technologies, Inc., Santa Clara, CA, USA).

### 4.4. Statistical Analyses

Each treatment was replicated 3 times, and the results were expressed as the mean ± standard error. The experimental data were processed using SPSS 24.0 software; a one-way analysis of variance was conducted for all data. Graphs were plotted using Origin 2024, with a significance level set at *p* < 0.05.

## 5. Conclusions

**Figure 12:**
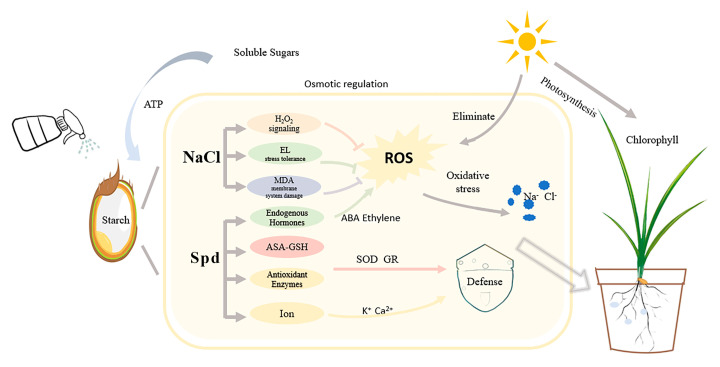
Response mechanism of rice seed germination and seedling growth to exogenous spermidine under salt stress.

Under NaCl stress conditions, seed priming with 0.1 mmol·L^−1^ Spd can enhance the germination rate of rice seeds, alleviate oxidative stress and cell membrane damage, reduce ionic toxicity, balance endogenous hormones, and thereby promote seedling growth. Increasing the net photosynthetic rate improves the chlorophyll content in rice seedlings. Furthermore, among salt-sensitive and salt-tolerant rice varieties, NaCl stress has a more severe negative effect on salt-sensitive rice varieties, and the application of exogenous Spd has a more significant alleviating effect on them. Therefore, Spd priming can stimulate the stress acclimation of seeds, promote seed germination, and mitigate the damage of NaCl stress on rice seedlings (Figure 12).

## Figures and Tables

**Table 1:** Response mechanism of morphological indices in rice seedlings to exogenous spermidine soaking under NaCl stress.

Variety	Treatment	Plant Height (cm)	Root Length (cm)	Leaf Area (mm^2^)	Stem Base Width (mm)	Aboveground Fresh Weight (g)	Underground Fresh Weight (g)	Shoot Dry Weight (g)	Dry Weight of the Root (g)
HD961	CK	46.20 ± 0.53 b	14.57 ± 0.47 ab	272.30 ± 8.13 a	2.70 ± 0.12 b	500.33 ± 9.53 b	202.33 ± 15.59 a	121.67 ± 0.88 ab	25.33 ± 2.33 ab
	S	51.23 ± 1.76 a	16.23 ± 0.89 ab	302.93 ± 37.00 a	3.37 ± 0.19 a	619.67 ± 49.83 a	220.33 ± 12.72 a	156.67 ± 22.64 ab	30.67 ± 4.63 ab
	N	40.30 ± 1.00 c	13.00 ± 0.58 b	306.63 ± 23.47 a	2.63 ± 0.09 b	448.67 ± 5.93 b	129.67 ± 5.55 b	101.33 ± 2.91 b	18.00 ± 2.08 b
	NS	46.97 ± 1.03 b	18.23 ± 2.11 ab	341.90 ± 19.63 a	2.97 ± 0.03 b	522.67 ± 16.19 b	233.67 ± 4.63 c	120.00 ± 11.50 a b	33.33 ± 3.28 ab
9311	CK	30.80 ± 1.80 a	14.37 ± 0.42 c	766.50 ± 21.42 d	3.23 ± 0.07 b	450.00 ± 15.63 b	213.67 ± 17.38 b	93.67 ± 3.33 d	24.00 ± 3.06 b
	S	34.33 ± 0.50 a	23.53 ± 2.08 a	853.27 ± 17.05 c	4.00 ± 0.17 a	591.67 ± 32.83 a	271.67 ± 4.70 a	129.00 ± 4.04 c	35.67 ± 0.33 a
	N	21.33 ± 0.44 b	14.17 ± 1.09 c	323.20 ± 24.30	2.17 ± 0.22 c	240.67 ± 23.35 c	142.33 ± 10.59 c	41.00 ± 7.21 b	16.33 ± 0.88 c
	NS	31.73 ± 3.51 a	19.47 ± 0.29 b	660.27 ± 23.72 a	3.33 ± 0.17 b	379.67 ± 28.9 b	295.00 ± 14.42 a	73.67 ± 3.84 a	29.33 ± 2.40 ab

Note: Different lowercase letters indicate significant differences at the *p* < 0.05 level among treatments within the same time point.

**Table 2:** Response mechanism of leaf ion content in rice seedlings to seed soaking with exogenous spermidine under NaCl stress. Different letters indicate statistically significant differences (*p* < 0.05).

Variety	Treatment	Na^+^ (μg/g)	Cl^−^(μg/g)	K^+^ (μg/g)	Ca^2+^ (μg/g)
9311	CK	14.05 ± 0.75 b	3638.16 ± 50.89 c	6012.38 ± 9.17 a	648.95 ± 7.06 b
leaf	S	8.37 ± 0.35 c	2402.62 ± 156.40 d	5793.4 ± 176.00 a	739.76 ± 26.24 a
	N	27.89 ± 0.68 a	7544.33 ± 178.36 a	2866.85 ± 88.62 c	395.75 ± 26.41 d
	NS	14.66 ± 0.30 b	4299.97 ± 18.78 b	4765.37 ± 50.21 b	544.91 ± 9.16 c
9311	CK	93.45 ± 2.07 c	1139.20 ± 44.77 b	972.48 ± 6.89 b	117.75 ± 1.67 d
root	S	62.01 ± 0.37 d	672.99 ± 34.41 c	1049.38 ± 9 a	163.42 ± 6.66 c
	N	428.96 ± 3.83 a	1818.45 ± 7.67 a	342.81 ± 2.75 d	213.14 ± 2.37 b
	NS	365.63 ± 1.63 b	1131.17 ± 6.65 b	372.19 ± 4.58 c	505.92 ± 4.13 a

## Data Availability

Data are contained within the article or Supplementary Materials.

## Supplementary Materials

The following supporting information can be downloaded at: https://www.mdpi.com/article/10.3390/plants13243599/s1.
