# Supplementary material for: Effects of Exogenous Spermidine on Seed Germination and Physiological Metabolism of Rice Under NaCl Stress
Source: Plants (Basel). 2024 Dec 23;13(24):3599. doi: 10.3390/plants13243599 (PMC11679135; doi:10.3390/plants13243599)
Supplement: Supplementary file 1 [file plants-13-03599-s001.zip › plants-3361336-supplementary.pdf]

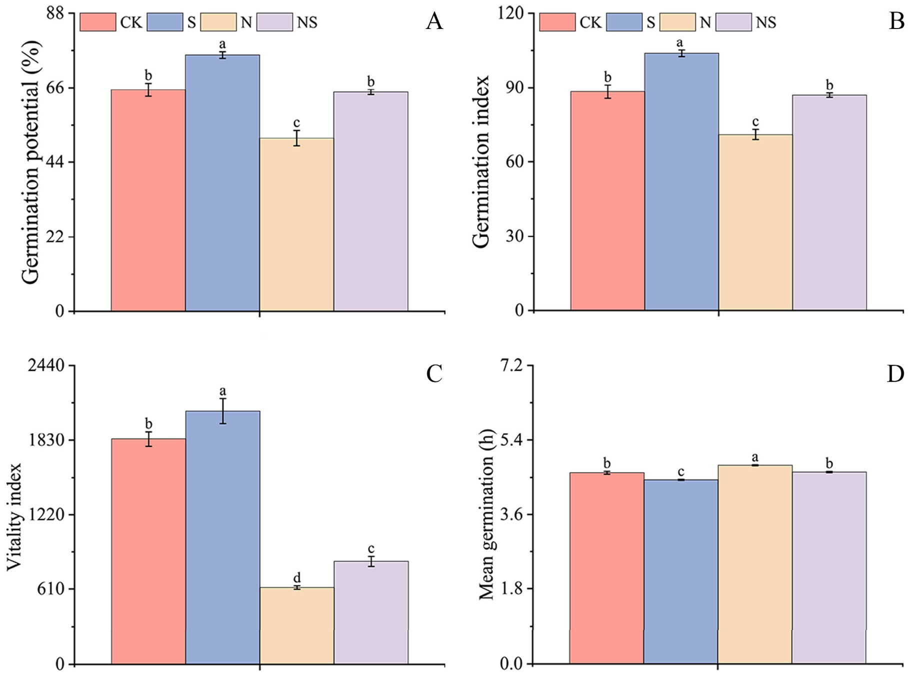

Figure S1. Response mechanism of germination potential (A), germination index (B), vigour index (C), and average germination time (D) of HD961 rice seeds to exogenous spermidine under salt stress. Here, S is spermidine seed treatment, N is NaCl treatment, NS is a combination of NaCl and spermidine seed treatments, and CK is no NaCl. In the following figures and tables, S, N, NS, and CK represent the same meanings. Different letters indicate statistically significant differences ($p < 0.05$).

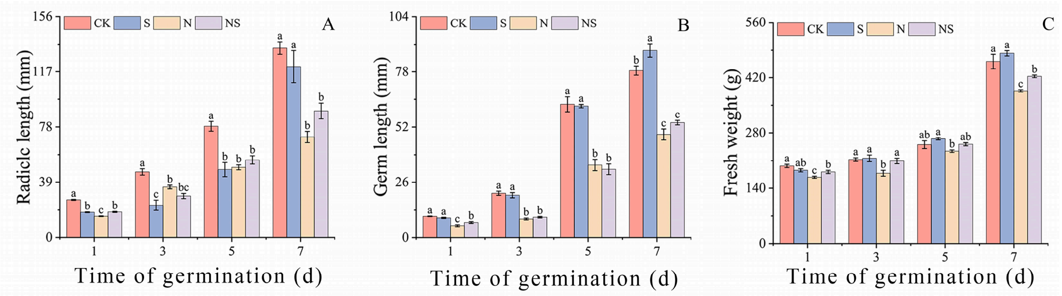

Figure S2. Response mechanism of root length (A), shoot length (B), and fresh weight (C) of HD961 rice variety to exogenous spermidine seed soaking under salt stress for 1, 3, 5, and 7 d. Different letters indicate statistically significant differences ($p < 0.05$).

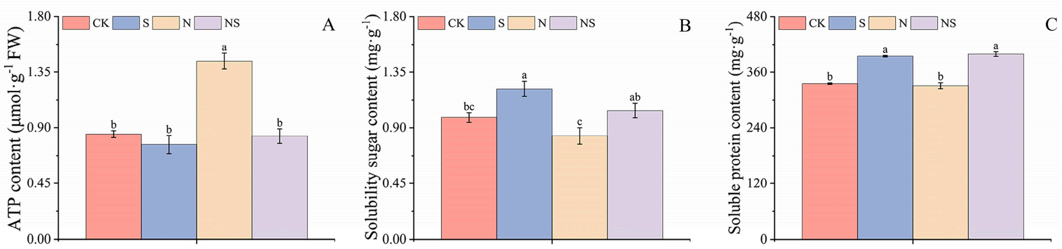

Figure S3. Figure 4. Response mechanism of ATP content (A), soluble sugar (B), and soluble starch (C) in HD961 rice variety to exogenous spermidine seed soaking under NaCl stress. Different letters indicate statistically significant differences ($p < 0.05$).

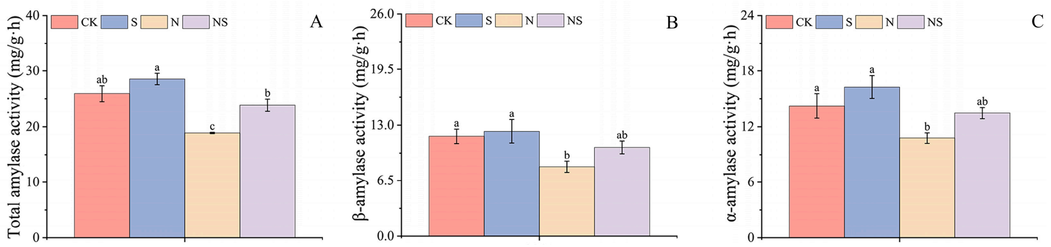

Figure S4. Response mechanisms of total amylase activity (A), α-amylase activity (B), and β-amylase activity (C) in the HD961 rice variety to exogenous spermidine soaking under NaCl stress. Different letters indicate statistically significant differences ($p < 0.05$).

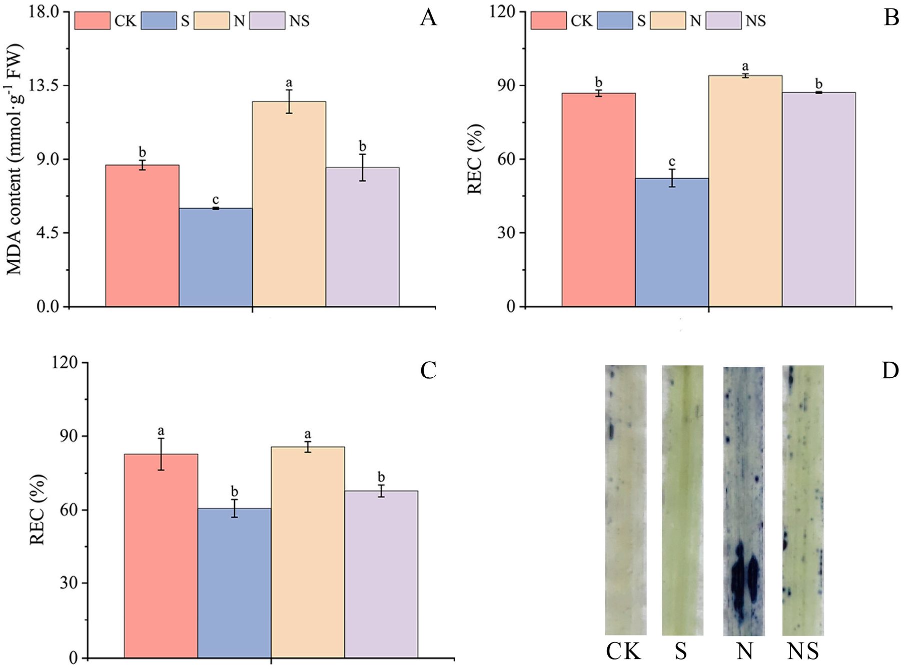

Figure S5. Influence of exogenous spermidine seed soaking on MDA content (A), electrolyte leakage in leaves (B) and roots (C), and H2O2 distribution in leaves (D) of HD961 rice variety under NaCl stress. Different letters indicate statistically significant differences ($p < 0.05$).

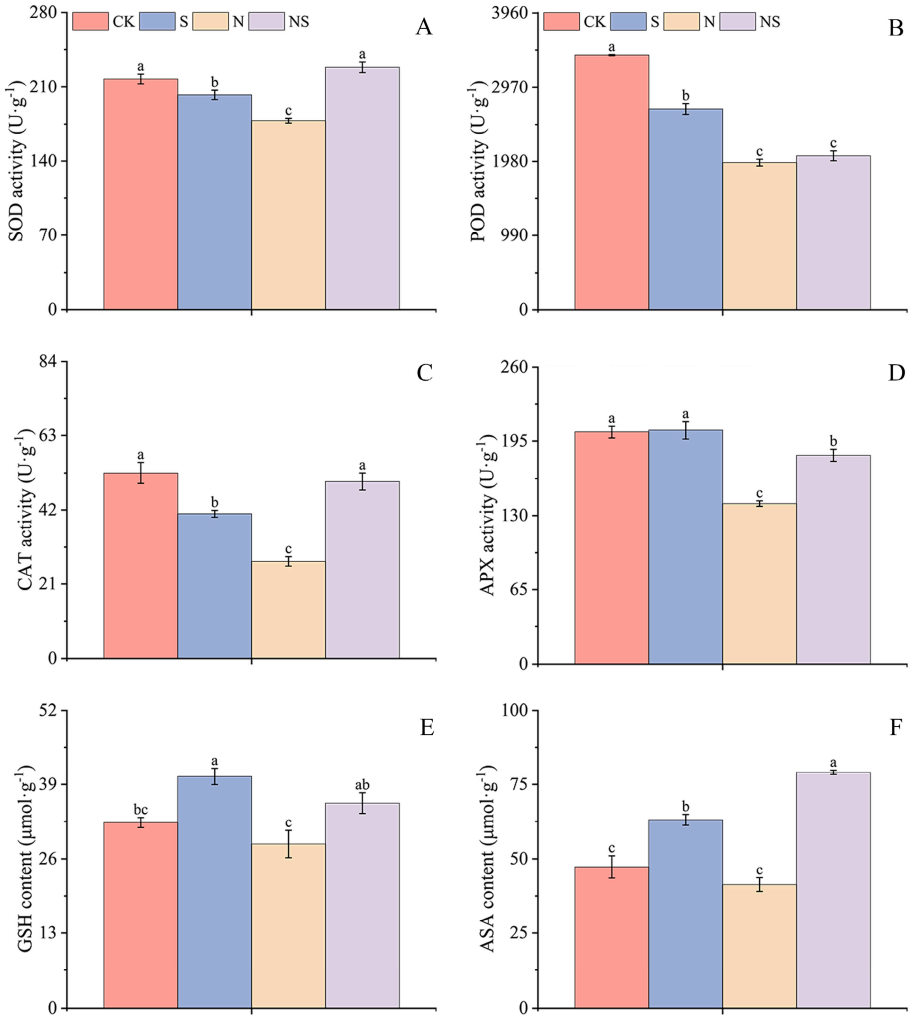

Figure S6. Response mechanisms of superoxide dismutase (A), peroxidase (B), catalase (C), ascorbate peroxidase (D), glutathione (E), and ascorbic acid (F) contents in HD961 rice seeds to exogenous spermidine soaking under NaCl stress. Different letters indicate statistically significant differences ($p < 0.05$).

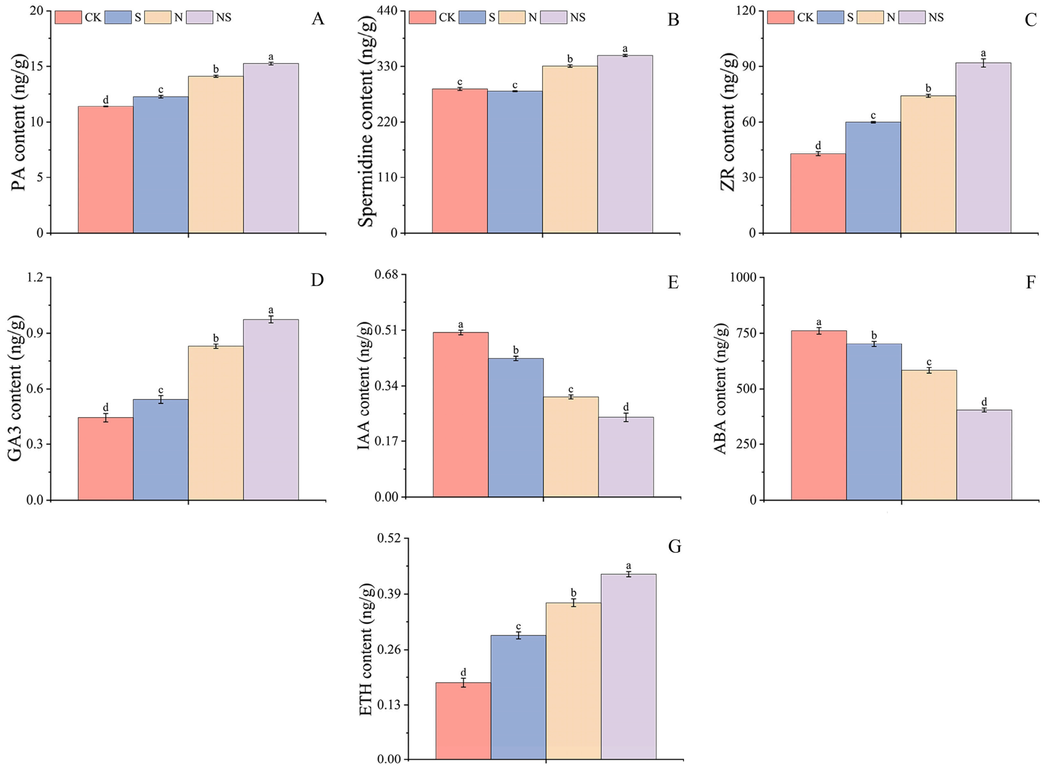

Figure S7. Response mechanism of polyamine content (A), spermidine content (B), zeatin content (C), gibberellin (D), auxin (E), abscisic acid (F), and ethylene content (G) in rice seeds to exogenous spermidine soaking under NaCl stress. Different letters indicate statistically significant differences ($p < 0.05$).

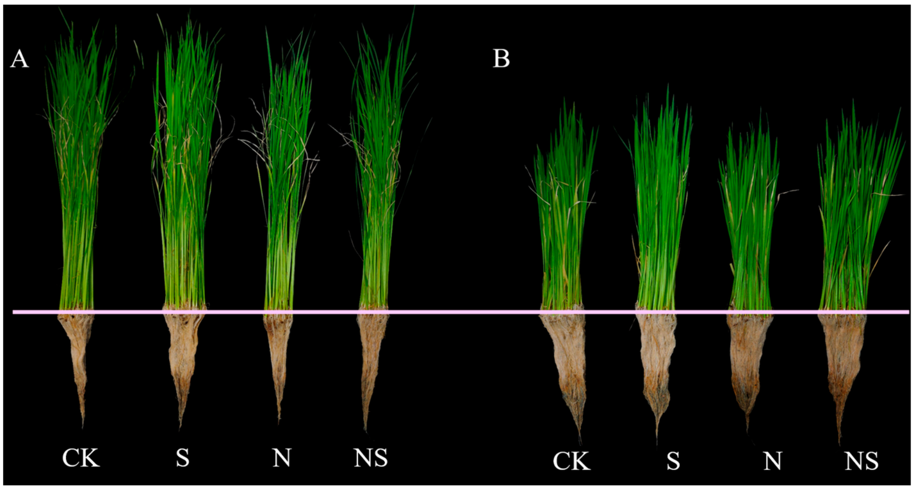

Figure S8. Response mechanism of rice seedling growths of HD961 (A) and 9311 (B) to exogenous spermidine soaking under NaCl stress.

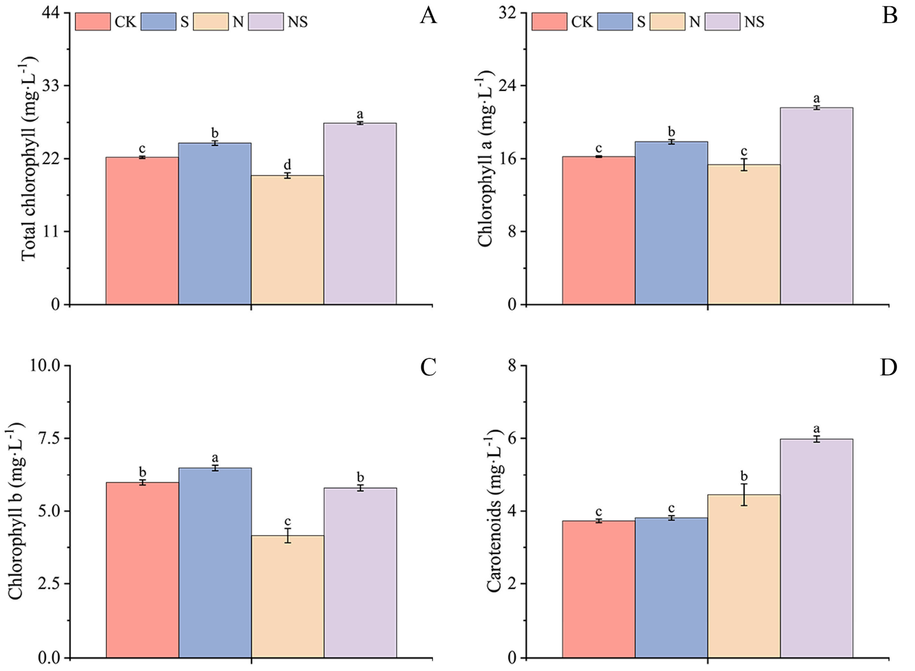

Figure S9. Response mechanism to exogenous spermidine soaking under NaCl stress of total chlorophyll (A), chlorophyll a (B), chlorophyll b (C), and carotenoid (D) contents of HD961 rice seedlings. Different letters indicate statistically significant differences ($p < 0.05$).

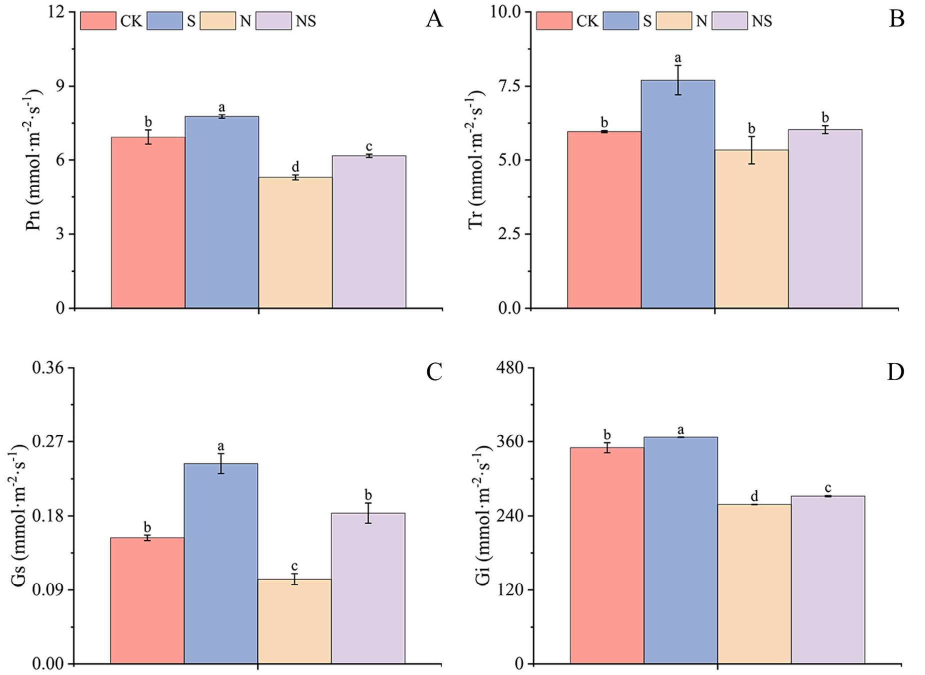

Figure S10. Response mechanism to exogenous spermidine soaking under NaCl stress of net photosynthetic rate (A), transpiration rate (B), stomatal conductance (C), and intercellular carbon dioxide concentration (D) of HD961 rice seedlings. Different letters indicate statistically significant differences ($p < 0.05$).

Table S1. Response mechanism of leaf and root ion content in rice seedlings to seed soaking with exogenous spermidine under NaCl stress. Different letters indicate statistically significant differences ($p < 0.05$).

Variety	Treatment	Na^+(μg/g)	Cl^-(μg/g)	K^+(μg/g)	Ca^{2+}(μg/g)
HD961	CK	9.13±0.50c	2435.11±169.06d	6099.95±66.59b	818.57±7.6b
leaf	S	18.80±0.31c	5114.18±40.40c	6851.14±27.04a	1012.56±13.15a
	N	1638.22±15.71a	11098.53±211.96a	2704.75±25.66d	383.76±14.46d
	NS	1295.11±7.91b	5604.42±35.00b	5192.70±51.79c	512.75±8.02c
HD961	CK	464.64±4.92c	1710.52±56.34c	998.62±10.35a	259.80±4.33c
root	S	342.87±9.22d	1442.45±28.65d	948.12±1.90b	316.74±2.32a
	N	952.49±4.80a	2655.40±60.38a	399.52±4.69d	213.32±3.72d
	NS	613.58±4.57b	2490.39±19.00b	534.17±0.36c	284.40±1.26b

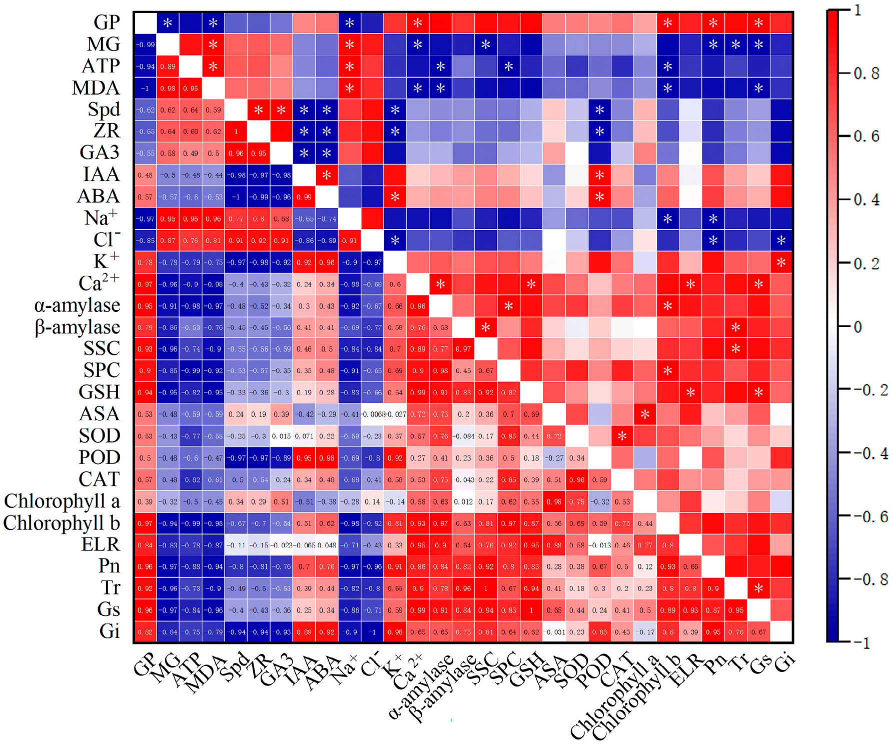

Figure S11. Response mechanism of rice seed germination and seedling growth to exogenous spermidine on HD961 and 9311 under salt stress. Red indicates a positive correlation between the two parameters, and blue indicates a negative correlation. *P≤0.05.
